# Giving voice to older adults living with frailty and their family caregivers: engagement of older adults living with frailty in research, health care decision making, and in health policy

**DOI:** 10.1186/s40900-016-0038-7

**Published:** 2016-06-17

**Authors:** Jayna Holroyd-Leduc, Joyce Resin, Lisa Ashley, Doris Barwich, Jacobi Elliott, Paul Huras, France Légaré, Megan Mahoney, Alies Maybee, Heather McNeil, Daryl Pullman, Richard Sawatzky, Paul Stolee, John Muscedere

**Affiliations:** 1 Department of Medicine and Community Health Services, University of Calgary, Calgary, AB Canada; 2 Canadian Frailty Network, Vancouver, BC Canada; 3 Canadian Nurses Association, Ottawa, ON Canada; 4 BC Centre for Palliative Care, Vancouver, BC Canada; 5 School of Public Health and Health Systems, University of Waterloo, Waterloo, ON Canada; 6 South East Local Health Integration Network, Belleville, ON Canada; 7 Department of Family Medicine and Emergency Medicine, Université Laval, Québec City, Québec Canada; 8 Canadian Frailty Network, Network for Centres of Excellence, Kingston, Canada; 9 Patients Canada, Toronto, ON Canada; 10 Faculty of Medicine, Memorial University of Newfoundland, St. John’s, NF Canada; 11 Trinity Western University, Langley, BC Canada; 12 Centre for Health Evaluation and Outcome Sciences, University of British Columbia, Vancouver, BC Canada; 13 Department of Critical Care Medicine, Queen’s University, Kingston, ON Canada

**Keywords:** Empowerment, Engagement, Family caregivers, Frail health care seniors, Partnerships, Policy, Research, Social care

## Abstract

**Plain English summary:**

The paper discusses engaging older adults living with frailty *and* their family caregivers. Frailty is a state that puts an individual at a higher risk for poor health outcomes and death. Understanding whether a person is frail is important because treatment and health care choices for someone living with frailty may be different from someone who is not (i.e., who is fit). In this review, we discuss strategies and hurdles for engaging older adults living with frailty across three settings: research, health and social care, and policy. We developed this review using published literature, expert opinion, and stakeholder input (including citizens). Engaging frail older individuals will be challenging because of their vulnerable health state - but it can be done. Points of consideration specific to engaging this vulnerable population include:

- In any setting, family caregivers (defined to include family, friends, and other social support systems) play an important role in engaging and empowering older adults living with frailty
- Engagement opportunities need to be flexible (e.g., location, time, type)
- Incentivizing engagement for researchers and citizens (financial and otherwise) may be necessary
- The education and training of citizens, health and social care providers, and researchers on engagement practices
- Patient-centered care approaches should consider the specific needs of individuals living with frailty including end-of-life care and advanced care planning
- Influencing policy can occur in many ways including participating at institutional, regional, provincial or national committees that relate to health and social care.

**Abstract:**

Older adults are the fastest growing segment of Canada’s population resulting in an increased number of individuals living with frailty. Although aging and frailty are not synonymous the proportion of those who are frail increases with age. Frailty is not defined by a single condition, but rather a health state characterized by an increased risk of physical, mental, or social decline, deterioration of health status, and death. Recognizing frailty is important because earlier detection allows for program implementation focused on prevention and management to reduce future hospitalization, improve outcomes, and enhance vitality and quality of life. Even though older adults living with frailty are significant users of health care resources, their input is under-represented in research, health care decision making, and health policy formulation. As such, engaging older adults living with frailty and their family caregivers is not only an ethical imperative, but their input is particularly important as health and social care systems evolve from single-illness focused to those that account for the complex and chronic needs that accompany frailty. In this review, we summarize existing literature on engaging older adults living with frailty and their family caregivers across three settings: research, health and social care, and policy. We discuss strategies and barriers to engagement, and ethical and cultural factors and implications. Although this review is mainly focused on Canada it is likely to be broadly applicable to many of the health systems in the developed world where aging and frailty pose important challenges.

## Background

Older adults are the fastest growing segment of Canada’s population, and the most vulnerable in terms of susceptibility to frailty [1–3]. Frailty is a state of increased vulnerability predictive of increased rates of adverse health outcomes and mortality; although frailty increases with age they are not synonymous. The growing number of older adults living with frailty is concerning given that current health care systems were mainly designed to address one medical issue at a time, many of which are considered reversible illnesses. The present system is ill-equipped to meet the specific, and often complex chronic needs of older adults living with frailty. Indeed, older adults living with frailty are extensive users of health and social care services and are greatly impacted by procedural and policy decisions across all settings of care. Barriers resulting from illness and frailty often prevent the elderly from becoming fully engaged in research surrounding their health, in setting priorities for health care decision making, or in policy decisions that inform their care. All of these factors influence health outcomes including the quantity and quality of life, so it is crucial that those directly affected have a voice in any decisions made.

Citizen engagement is an ethical imperative that embraces the principles of inclusivity, mutual respect and co-design [4]. Furthermore, citizens are the experts in their own lives and provide a unique perspective on the values and priorities of the communities in which they live. Evidence from Canada, the United Kingdom (UK), and the United States (US) shows that engaging citizens in health care decision making improves the patient experience, improves health care, and contributes to more appropriate funding and administrative decisions [5–8].

### Framing frailty

Frailty can be conceptualized as a state marked by an accumulation of physiological deficits over time [9]. These accumulated deficits increase an individual’s vulnerability to poor health outcomes including physical, mental or social decline, and death. For an individual living with frailty, even fairly minor health events can trigger major changes in an individual’s health status. Frailty can occur as the result of a range of diseases and medical conditions, and is manifested by alterations in function including reduced walking speed, weight and muscle loss including decreased grip strength, chronic fatigue, loss of physical activity, and memory loss [1]. It is important to note that frailty is not an inevitable part of aging, and that care decisions for older adults living with frailty may be different from those who are fit or those who are younger or fit [10]. Indeed, the progression of illness in an individual with frailty is distinct from someone who experiences an acute illness [11]. When individuals are recognized as being frail, interventions and treatment plans can be tailored to their specific needs. By recognizing frailty, health and social care providers can implement programs focused on early detection, prevention, and management to reduce future hospitalization, improve outcomes, and enhance vitality and quality of life.

### Defining citizen engagement and the family caregiver

While there are many definitions of citizen (public) engagement, we have chosen to adopt, with revisions, the definition first articulated by the Canadian Institutes of Health Research (CIHR) [12]:

*Citizen engagement is the meaningful, timely, and appropriate involvement of individuals and potential support systems in policy development, program planning and implementation, research development, and health care decision making. In the context of health and social care, engagement can occur in the research setting, health and social care setting, and policy setting.*

“Citizen” refers to all people who formally or informally use health care services. For brevity, “family caregiver” refers to family, friends, neighbours, and other social support systems of older adults living with frailty. A summary of some of the definitions used in this paper is included in Appendix 1.

This definition of citizen engagement underscores the essence of engagement as a dynamic and meaningful process that goes beyond passive consultation. It seeks to actively and continuously engage, empower, and partner with citizens in the research, health care decision making, and policy development that will impact them. Theories of citizen engagement generally ascribe a spectrum of engagement with increasing levels: communication (information is communicated to citizens or caregivers); consultation (information is collected from citizens); or participation (citizens are partners in an exchange of information and deliberation with others through collaborative efforts) [13, 14]. Successful citizen engagement requires careful planning and strategic approaches. Strategies may be diverse at different points in time. To be successful, citizen engagement needs to adapt to the condition, state, capability, and background of the individuals being engaged, but also to the ultimate goals of the engagement process (e.g., Carman et al. [6]).

Several Canadian and international organizations have formal plans and strategies for patient engagement in research and health care. Examples include Health Canada and CIHR’s Strategies for Patient-Oriented Research (SPOR) in Canada, Patient-Centered Outcomes Research Institute (PCORI) in the US, the National Institute for Health Research’s INVOLVE in the UK, and the international James Lind Alliance [15–19]. While these extensive strategies offer an excellent starting point, engaging older adults living with frailty and family caregivers has specific barriers and challenges that require special consideration as we engage and partner with this particularly vulnerable group. Further, extra efforts will be needed to engage at risk individuals based on socio- and geo-demographics including first nations, immigrants, low education, low economic status, and individuals living in rural settings.

### Content overview and conceptual framework

A family-centric model of care emphasizing the role of family caregivers is particularly relevant to engaging and empowering older adults living with frailty. Given that evidence surrounding best practices for engaging this population is sparse, this review comprises relevant strategies for engaging adults and senior populations. Barriers and gaps in our understanding of engaging older adults living with frailty and their family caregivers, along with ethical considerations, are also discussed. For the purpose of this review, we use “health care” to refer to services provided across different settings (e.g., institutional and community), and by different providers (e.g., doctors, nurses, social workers, and occupational and physical therapists) that addresses the full spectrum and diversity of the needs of older adults living with frailty.

Engaging older adults living with frailty and family caregivers requires consideration of the following settings:

1. research designed to inform the care of older adults living with frailty; both in its conduct and in priority setting;
2. health care decision-making and individualized care planning across all settings of care; and
3. planning and policy of health care systems.

For the sake of clarity and conceptualization, these engagement settings have been separated in the present review. Meaningful engagement of older adults living with frailty and family caregivers will require a shared responsibility where citizens, research teams, health care providers, and policy decision makers are responsive to each other [20, 21]. Although this review is mainly focused on Canada it is likely to be broadly applicable to many of the health systems in the developed world where aging and frailty pose important challenges.

## Review

### Methods

The work presented in this paper was informed by recent realist syntheses on patient engagement by co-authors (Stolee et al., 2015; Elliott et al., 2016; McNeil et al., 2016) and supplemented by selected literature for topics not covered in the realist synthesis. As such, the design of this paper was not intended to be a formal systematic review, but one that was informed by the most relevant literature including systematic reviews, expert opinion, and stakeholder input, and could be described as a mixed methods review, or a literature review with a narrative synthesis. This type of review is within the typology of review types described by Grant and Booth [22]. The mixed methods review style was selected to create a broader, more applied description of engaging older adults living with frailty in decision making. It goes further than a traditional review in that it integrates the results of a participatory consultative process, which included patient representatives. The stakeholder input was gained via a Canadian Frailty Network (CFN)-sponsored participatory meeting and an online discussion board, as described below.

The Participatory meeting, *Giving Voice to Frail Elderly Canadians*, was coordinated with assistance from CFN’s Citizen Engagement Committee, and took place on September 27, 2015 in Toronto, ON, Canada. In preparation for the meeting, a draft version of this paper was prepared and distributed to all invitees of the meeting. The intent of the meeting was to have a collaborative discussion about engaging older Canadian adults living with frailty in research efforts, alongside expert opinions from across the healthcare continuum, including: older adults, caregivers, citizen advocacy representatives, researchers, health care professionals, policy makers, and industry partners. The distribution of participants at the meeting by the group they identified with was as follows: 6.8 % advocacy group representatives, 18.2 % citizen or volunteer caregivers, 18.2 % health care providers or administrators, 6.8 % policy persons, 40.9 % researchers, and 9.1 % other.

Briefly, the multidisciplinary group of approximately 60 participants actively discussed strategies to, and barriers of, engaging this vulnerable population using the paper as a guide. The voices of patients were represented through patient presentations which were given at the participatory meeting, and by older adults and family caregivers who participated in World café discussions used to develop key themes and statements for this paper. This involved providing participants and patient representatives with the opportunity to discuss the key themes and statements and rate them by indicating their level of agreement with statements generated during the discussions using electronic clickers. The statements generated during the meeting are included in Appendix 2. The discussions and statements with which there was agreement from the majority of the group were used to inform revisions to the draft manuscript. The revised manuscript was then posted on an online discussion board, which allowed 180 (including those who had participated in the face to face meeting) invited members, including patient representatives, to comment and provide feedback. As a result, the production of this paper involved a combination of a number of diversified voices, including that of our patient representatives. They have been given the opportunity to both directly and indirectly contribute to the development of this paper through the generation of the themes and topics discussed, and by providing direct feedback on the original draft of this paper. Further and of note, one of the authors of this paper is a member of CFN’s Citizen Engagement Committee who is also a caregiver for an older adult living with frailty.

### Engagement in research priority setting and conduct

Engagement in research refers to active involvement and partnership in the research process. This includes determining which research questions should take priority, conducting research (e.g., development of research design and methodology, interactions with research participants), and communicating and disseminating research findings. Patients and family caregivers can have an important role to play in research surrounding their care and it has been recognized that “the knowledge, wisdom, and energy of individuals and families” is an unexploited resource for health care improvement [23]. Demonstrated benefits of citizen engagement in research in priority setting include:

- improved research quality (i.e., improved research questions, hypotheses, and methodologies) [24–28];
- improved relevance of conducted research [29, 30]; and
- avoiding waste in research [31].

Other hypothesized benefits that require thorough evaluation include [8, 31, 32]:

- assurance that funded research reflects the needs and values of public funders;
- increased translation of research findings into policy and potential to positively influence the uptake of research results, causing meaningful changes; and
- increased public confidence and understanding of the research process.

Engaging vulnerable communities such as older adults living with frailty is especially important since data for these is often limited or missing [6, 33, 34]. More specifically, research often focuses on single-conditions, and often excludes individuals who have multi-morbidities and those who are elderly, which is common in older adults living with frailty. The generalization of research priorities and findings from fit individuals to older adults living with frailty requires data and input from this population and their family caregivers. However, there has been limited research focusing on engaging older adults in research and research priority setting. McNeil and colleagues [35] addressed this issue with a realist synthesis [36] of available peer-reviewed and grey literature focusing on why, how, and in what context older adults are engaged. This synthesis, conducted in partnership with older adult participants resulted in the identification of the following four principles and strategies for engagement.

First and foremost, the older adult must be a central consideration, which should be a guiding principle of research teams and institutions aiming to engage citizens [37, 38]. Meaningful engagement necessitates a holistic approach that acknowledges the citizen’s characteristics, demographics, and their social support network. The research team needs to discuss the older adult’s preferences, goals, needs, and expectations for engagement in health care research [39, 40]. Additionally, the citizens should be composed of a diverse and representative sample of the elder population [41], which will also include the different levels of engagement preferred by participants.

Secondly, the skills, characteristics, attitudes, and experiences of research teams play a role in how, to what extent, and the duration citizens and their family caregivers are engaged in health care research. As participating in research with little to no experience can be intimidating, it is important for the research team to be mindful of the attitudes they portray to citizens. Negative attitudes and experience of research teams may create barriers to meaningful engagement and limit ongoing and future partnerships [42]. Additionally, a recent poll suggests that citizens may be interested in being engaged, but may not know how, and by what process to do so [43]. The institutional paradigm from researchers to administrators needs to encourage a culture shift toward meaningful engagement [44].

Third, the research method chosen will have an effect on areas of opportunity for citizen engagement. To enhance such opportunities, flexibility and being open to change where it is possible, are important. Where engagement opportunities arise is also important: for example, a “check box” approach at the end of a project when submitting a paper or policy report results in tokenistic involvement. Rather, engagement should be thoughtful and interactive, with older adults engaged in the design process at the beginning of a project, and involved as much as possible throughout the course of the project [45].

Finally, knowledge translation and dissemination of research results present opportunities for successful and continued engagement. Discussion and partnership between researchers and citizens are important for dissemination of research allowing for a collaborative effort in determining the preferred format of information and information exchange [46].

### Challenges to engagement in the research setting

A major challenge to engagement in research is the power imbalance, arising from different levels of knowledge or decision making authority, which may exist or be perceived to exist between researchers and citizens. Ultimately, these require researchers to share their decision making authority or knowledge which if not done can lead to co-option or tokenistic engagement efforts causing mistrust among patients and family caregivers [47]. Past experiences shape expectations and influence participants’ willingness to engage in research [48, 49]. One step investigators can take to overcome this barrier is asking participants about their preferred level of engagement (from passive to active roles) [50] and plan accordingly. Other barriers to participating in research include poor health, lack of perceived benefit, and distrust of research staff. Engaging family caregivers in the process may be one strategy to help in recruiting and retaining older adults living with frailty in research studies who have specific challenges [51].

Locations where participants are being engaged need to be accessible to those with mobility impairments or other challenges [48, 52]. The scheduling of engagement opportunities should be convenient for older adults [53], with transportation being a key consideration [47]. Web-based communication platforms (e.g., Skype) or online discussion boards are alternative solutions when in-person meetings are not possible. In Canada, there are many examples of successful citizen engagement efforts with older adults. Examples include advisory groups such as the Ontario Dementia Advisory Group which is made up of individuals with dementia and caregivers [54] with a motto “nothing about us without us” encompassing the goal of the group to be involved in whatever way possible in decisions that will affect their lives; citizen juries [55]; participant pools such as the Seniors Helping Advance Research Excellence (SHARE) group at McMaster University (Gilbrea Centre) [56] which facilitates opportunities for seniors to volunteer in various research projects at the university; and research partnerships with older adults and their caregivers such as the Seniors Helping as Research Partners (SHARP) group based out of the University of Waterloo [57], which aims to advance the development of research priorities, collaborations and improvement of the health care system for older adults.

Adding to the challenges with citizen engagement is the large gap in the literature on how to combine community collaboration and active engagement with research methodology [27]. In addition to limited guidance, systemic constraints may also lead to missed engagement opportunities or ones that are not meaningful. From the perspective of the researcher, there are few incentives for researchers to engage citizens, making the time and costs associated with engaging citizens major barriers in the engagement process, as described by researchers at the CFN citizen engagement meeting. One possible solution is for granting agencies to change funding guidelines such that they require and fund citizen engagement. For instance, granting guidelines could specify the required engagement, and resources available to those applying for funds [58]. Furthermore, providing funding to both citizens and researchers for projects on *how to* engage will help fill the gaps in the literature and this should be addressed by funding agencies.

Many of the aforementioned strategies and barriers are based on evidence on engaging older adults [35]. It is less clear whether the same strategies are suitable for meaningful engagement of older adults living with frailty and/or family caregivers. Given the limited evidence for this large and growing population, additional research is needed to refine and test specific strategies for partnering with this heterogeneous and vulnerable group of citizens in health care research.

### Engagement in the health care setting: health care decision making and individualized care planning

Engagement is integral for person-centred care in which individuals’ unique needs, concerns, and expectations take priority in health care decisions that inform care [59]. Person-centred care is about delivering the right care to the right individual at the right time and in the right place [60, 61]. One of the key aspects of engagement in health care settings is to move away from provider-led care and towards empowering older adults living with frailty and family caregivers to make their own decisions regarding the frail older adult’s care. Engagement and empowerment can and should occur across the continuum of care, from community based to long-term care settings. National health care organizations such as the Canadian Nurses Association (CNA), the Canadian Medical Association (CMA), the College of Family Physicians of Canada (CFPC), and Health Action Lobby (HEAL) have a longstanding commitment to advancing person-centered care that is seamless along the continuum of care [60–64]. This movement is grounded in the values and principles of primary health care as outlined in the World Health Organization’s 1978 Declaration of Alma-Ata that the needs of patients and their families must be the main drivers of health care delivery [65].

Further, person-centred care for older adults living with frailty not only involves the development of mutually developed care plans and decision making, but also requires moving away from a disease-specific model of care to one that focuses on care plans that focus on the optimal treatment for the well-being of older adults living with frailty. Engaging older adults living with frailty, who have multi-morbid conditions is important for further disease prevention and self-management, and will ultimately lead to an increased chance of improved care and well-being [66]. Playing a more active, engaged role in health care can improve patients’ quality of care and health outcomes, especially considering the expertise of the patient with respect to their own illness and life situation [67, 68]. To improve care and outcomes for this population, older adults living with frailty and their caregivers need to be engaged as *active* partners in decisions relating to their health care.

Moreover, health care providers’ knowledge base needs to include geriatric knowledge and skills in order to ensure provision of person-centered care to older adults living with frailty. For example, a comprehensive understanding of the social determinants of health includes an understanding of needs relating to social, physiological, spiritual, and/or cultural aspects. This understanding of need addresses where the older adult living with frailty resides, what supports they have, their health status, culture, and values/beliefs. Person-centered care delivery requires building and empowering capacity of patients and their family caregivers. Strategies that create an environment for citizen engagement in a person-centred care context include [69]:

- creating an environment where older adults living with frailty and family caregivers feel safe and comfortable during healthcare interactions;
- building a relationship on mutual trust and respect among older adults living with frailty, family caregivers, and health care providers;
- conducting comprehensive health and social assessments;
- addressing a variety of determinants (e.g., psychosocial; physical, cognitive, environmental) of health which are ethically and culturally sensitive;
- providing respite for family caregivers;
- providing leadership, training, and education for health care providers, patients, and family caregivers about how to best to engage in a meaningful partnership; and,
- sharing information between health care providers, patients, and social support networks.

Ongoing communication between care providers and older adults living with frailty about preferred level of engagement in decisions that inform their care is also extremely important. In the health care setting, all parties will gain knowledge, skills, and experience with time, which may influence the preferred level of engagement. Additionally, older adults living with frailty have changing health needs, which may alter their ability and desire to engage. Similarly, the family caregiver may choose to alter their level of engagement fitting with the current health needs of the individual. This is particularly true for individuals who are in late-life or nearing end-of-life; nonetheless, the majority of patients still appreciate the chance to discuss end-of-life care [70].

Quality end-of-life care is particularly important for older individuals living with frailty [71]. Yet, accessing quality end-of-life care is a significant problem in many provinces across Canada, and there is little literature on palliation for these individuals, as opposed to those who have better defined life-limiting diseases such as a cancer diagnosis. The involvement of older adults living with frailty and their caregivers in developing their care plans is crucial to allow for care that matches a patient’s wishes, values, and beliefs. In addition, their involvement would help remobilize social and health care resources to allow healthcare professionals to provide the desired options to patients and family caregiver. Moreover, it may galvanize researchers to provide more evidence to inform their care.

Because of the changing needs of older adults living with frailty and the delivery of care by multiple care providers, there are often inconsistencies in the care being provided. This often occurs, for example, for those receiving home care where there can be a disconnect between the quality and quantity of care [72, 73]. This disconnect has also occurred by moving nurses to task-based practice (in an attempt to serve more clients) without having adequate resources to meet the demand. Also lacking are infrastructures that provide care providers with the data, feedback, or other mechanisms to ensure that all providers and the patient are aware of the outcomes of care delivered. These barriers can be addressed by:

- supporting the patients during transitions within or between care settings;
- sharing information for seamless coordination of care;
- collaboration of health care providers within and across care settings to provide efficient and effective care; and,
- collaboration of health and social disciplines with a broader number of disciplines: engineering, architecture, urban studies, administration, religious studies, arts, etc.

Prince Edward Island is an example of a Canadian province in which health care in the home and long-term care sector has shifted from a task-based model to one that is person-centered [74]. A model was implemented that increases partnerships with older adults living with frailty and their family caregivers, recognizes their needs, and plans care around their needs, wishes, and choices. As part of this shift, long-term care residences are being renovated to “households” of 12–14 people with private rooms and washrooms, and a shared kitchen and dining area. Additionally, rather than continuously changing staffing assignments, staff are being assigned to a specific households, which aims to improve trust and communication between care providers and residents. This shift has incented care providers and administrators to change their values and philosophies of care away from the provider-led perspective to a person-centered, citizen-engaged, and team-based approach [74].

### Engagement in influencing health care system planning and policy

Citizens often report good clinical care, but poor treatment from the health care system [75]. The health status of older adults living with frailty means they will visit, often repeatedly, multiple medical and care settings within our health care system. These services often are not well linked or coordinated, contributing to the stress and pressures that patients, caregivers, and health care providers experience.

The overarching direction of health care systems is set by planning, development, implementation, administration, and evaluation of public policy and health care programs by governments, health care agencies, and not-for-profit advocacy organizations. Health policy change is an essential condition to redressing fragmented care and quality issues and other limitations of our health care systems. In the context of this review, we define health policy as setting a direction and providing resources for action to protect, promote, and restore mental and physical well-being [76]. Since health policy is intrinsically public, many factors come to bear across many sectors of society and involve communities, elected representatives, and governments working together. Citizen engagement is a reflection of a health community’s desire to participate in shaping public policy to achieve desired outcomes.

Provinces have used strategies that aim to engage patients in improving care administration, such as Ontario’s Health Links program, which aims to improve care for seniors and individuals with complex conditions by improving coordination of care [77]. Similarly, seven hospitals in Eastern Ontario recently undertook a major planning effort to improve access to high quality care across the region [75]. In addition to this initiative being guided by evidence where available, it is informed by external expert opinion including clinical input and patient advice [75].

Patient advisory boards and patient and caregiver participation on general advisory boards are becoming common strategies for the patient and caregiver voice to affect change at the level of hospital administration. The Kingston General Hospital (KGH) in Kingston, Ontario has served as model where patients are engaged in all aspects of health administration. Led by the Patient and Family Advisory Council (PFAC), Patient Experience Advisor citizen volunteers partner with KGH staff to provide direct input into policies, programs, and practices that affect patient quality of care and services [78].

The influence of citizen engagement on policy setting and planning can be exerted in different, often indirect ways. The first is by increasing citizen engagement across non-governmental organizations (NGOs). Older adults living with frailty and their family caregivers can influence policy and direction of the health care system by serving on working groups and committees for patient and caregiver advocacy organizations, research-oriented organizations (e.g., CFN), policy oriented organizations (e.g., Canadian Foundation of Healthcare Improvement) and individual research groups that are funded by these organizations [79–83].

Large-scale initiatives and alliances can drive health policy change at the provincial level by promoting citizen involvement in research. Alliances between advocacy and research organizations can influence policy by having one common strategic plan for health-based research. One such alliance is the Canadian Health Services and Policy Research Alliance (CHSPRA) that was formed as an outcome of the CIHR’s Institute of Health Services Policy Research (IHSPR) Initiative. The IHSPR Initiative supports health and policy research that aims to inform and evaluate effectiveness of the health care system. The IHSPR has developed a Canada-wide vision and strategy for health sciences and policy research. This initiative funds research that ultimately leads to evidence-based policy change to improve health care. Another Canadian example is the national Canadian Institutes of Health Research (CIHR) Strategy for Patient-Oriented Research (SPOR), which allocates funds to each province to develop Support for People and Patient-Oriented Research and Trials (SUPPORT) Units. These SUPPORT Units are teams of citizens, researchers, policy makers, funders, and health care professionals that aim to improve and support person-centered research, knowledge translation, and implementation of evidence across Canada. While these organizations are not specific to frail populations, frailty networks and research groups can collaborate with larger initiatives to influence policy change via the aforementioned avenues.

Advocacy organizations also influence policy change by improving dialogue between citizens and policy makers and acting as one voice. Indeed, organizations can relay important health issues surrounding health care that are important to the users. Citizen advocacy and engagement in health system direction setting is most effective when knowledge exchange is in place. Knowledge translation and mobilization are important strategies to building the requisite capacity and motivation of a health community to leverage influence. For example, improving public access to research and reports allows citizens and their elected representatives to define important research and health care priorities (e.g., James Lind Alliance) [18, 84, 85]. Several initiatives, including nationally funded programs have internet-based systems that make health care related research findings and other reviews accessible and understandable. An example is CIHR and McMaster University’s Evidence-Informed Health Care Renewal (EIHR) Portal, which contains freely available documents relating to health care policy [86]. Alternatively, other organizations have other resource platforms that include broad topics surrounding engagement (e.g., Canadian Foundation for Healthcare Improvement’s Resource Hub, and National Institute for Health Research’s Training and Support Resource) [87, 88].

Fortunately, considerations (e.g., strategies and barriers) to engaging older adults living with frailty and their family caregivers in the policy setting may be very similar to those observed for engagement in research and health care system contexts, given that this is the case with engaging older adults [35].

### Engaging family caregivers

Given the physical and cognitive limitations that can accompany frailty, these individuals often rely on family caregivers to help them navigate through the health care system. In Canada, there are an estimated four million Canadians caring for older family members [89]. As such, we need to focus on appropriately engaging both older adults living with frailty and their family caregivers.

Engaging social support systems is challenging since many family caregivers are stretched beyond their capacity and have high levels of stress [90]. Caregivers often experience psychological distress, especially as the number of hours spent providing care increases [91, 92]. Therefore, strategies to support older adults living with frailty should also consider interventions that are targeted towards optimizing the health and well-being of their family caregivers. These types of caregiver-focused interventions are more likely to be successful if they involve caregiver input in their development [73, 93].

Balancing the engagement of older adults living with frailty with that of their family caregivers can be challenging. Barriers specific to empowering older adults living with frailty in care decisions can include health care providers and family caregivers not facilitating their participation, and their dominance in decision making without the inclusion of input from the older person living with frailty [94]. Family caregivers can have particular difficulty with location of care and end-of-life discussions [73]. They may be unwilling to accept their frail relative is near the end of life or wish to protect them from potentially upsetting discussions [70]. They may wish to, move them outside of their home to other locations of care without adequate input from the affected individual [73].

Person-centered care and the development of care plans include the fundamental belief that every individual has the capacity, skills, competencies, and potential to assume responsibility for their health [95]. In the case of older adults living with frailty this may not be true. Potential barriers to engaging older adults living with frailty include lack of time, will, energy, or cognitive capacity to be actively involved in their health care decisions [96]. Furthermore, they may not necessarily have family caregivers who are available or able to help guide their decisions. Indeed, the same can be true when engaging this population in research or policy settings.

### Ethical considerations

There are many ethical and cultural aspects of working with older adults living with frailty that need to be taken under consideration when trying to engage this population [42]. As noted earlier in this document, “… in a democratic society, citizen engagement is an ethical imperative which embraces the principles of inclusivity, mutual respect and co-design”. While recognizing this imperative is an important first step, engaging older adults living with frailty, their family caregivers, and their communities can present many ethical challenges.

Perhaps the greatest of these challenges arises from the generally ageist culture in which we live, which can manifest itself in policies and practices that impinge efforts to engage older adults living with frailty. Recently, a researcher reported frustrations with regard to a project that was investigating the financial literacy of people in the 55–75 year age range. The Research Ethics Board at her institution asked what measures were being taken to ensure that research participants were not cognitively impaired. The implicit assumption was that once an individual reaches a certain age, they were likely to be cognitively impaired as to be competent. Although it is true that the incidence of cognitive impairment is higher in older populations, the reality is that the vast majority are competent to speak for themselves and to make informed decisions. Respectful engagement requires assuming competence as the default, and addressing each individual accordingly. While it may at times be more convenient to speak with a family member or other care provider, we must guard against assuming that physical frailty includes emotional and cognitive frailty as well.

Related ethical challenges in the context of health care decisions can arise regarding an individual’s choice to live at risk. Often family members and care providers seek to protect their loved ones living with frailty by moving them from the community into institutional care, both out of concern for their physical well-being and to reduce their own worries and anxieties [73]. It is not unusual for some individuals living with frailty to resist institutionalization. Clearly competent individuals can choose to live at risk providing they do not put others at undue risk in the process. However clinicians are not mere observers of such caregiver/care-recipient dynamics; the nature of their engagement can make them part of that dynamic as well. How do we balance the physically frail but mentally competent individual’s choice to live at risk in the community against the added stress and anxiety of their caregivers, while avoiding being co-opted by either party into supporting one position over the other? And, how do we balance the well-being of caregivers versus that of older adults living with frailty?

The converse can arise when encountering an older adult living with frailty who appears to lack appropriate family or other caregiver supports. What responsibilities do researchers have if they perceive an individual to be at risk? What if they suspect physical, emotional, or financial abuse? While we should guard against using such exceptional scenarios to set the general rules for how to go about engaging with older adults living with frailty, clinicians and researchers should be aware of such possibilities in advance and have contingency plans in place.

### Directions moving forward

It is important to note that citizen and patient engagement itself is a practice that is far from being perfected at this point in time [27, 42]. Older adults living with frailty are a diverse group of patients that exhibit physical and/or cognitive impairments, which may greatly hinder their ability to engage in research and decision making. Although these also pose challenges for engaging other vulnerable populations, in older patients living with frailty there may be other distinct barriers such as increased deferral to health care providers and decision makers, lack of familiarity with computers, shortened life span reducing continuity and progressive loss of function. The engagement of older adults living with frailty is relatively novel and one that still requires further research to inform its optimal practice. A valuable step in the process of the necessary culture shift would include educating all stakeholders on the benefits of engagement, and potential strategies to engage older adults in research. Establishing a relationship between all stakeholders involved in health care system including researchers is crucial to citizen engagement, with trust, role clarity, and communication all being essential components.

The aging Canadian population as in the rest of the developed world underlines the urgent need for ensuring the meaningful engagement of older adults living with frailty and their family caregivers in research, health care, and policy making across all settings of health care. Although many barriers and impediments can arise or have been described, exciting initiatives across Canada and abroad are providing evidence that these can be addressed effectively. For example, Health Canada’s advisory panel on health care innovation identified five areas for health care innovation [4] with one of these being patient engagement and empowerment. This is a hopeful sign of a cultural shift starting with decision makers that will percolate to all levels of the health care system.

## Conclusion

While engaging older adults living with frailty in decision-making does present challenges, our review suggests that engaging this population is feasible, the challenges can be overcome, and the effort is worthwhile. Although the quality of contribution from older adults living with frailty is not yet well-defined older adults living with frailty and their family caregivers still need and deserve to have a say in activities that inform their care. This engagement must be formal and embedded within all levels of healthcare including research, interactions with health care providers and policy setting. The resultant coordinated, person-centred care for older adults living with frailty will be more effective in the longer term for both for the individual and our society at large. Successful engagement of this vulnerable population must, however, balance the needs of older adults living with frailty and their family caregivers. Ultimately, engagement will result in and sustain positive change for the care and well-being of older adults living with frailty. To reach the full potential of engaging frail adults living with frailty, improving and evaluating methods to engage this population are required and we hope this review will encourage and support these efforts.

## Appendix 1

### Definitions

**Citizen**: all people who formally or informally use health care services in Canada.

**Citizen engagement**: meaningful, timely, and appropriate involvement of individuals and potential support systems in policy development, program planning and implementation, research development, and health care decision making. In the context of health care, engagement can occur in the research setting, health care setting, and policy setting.

**Family caregiver**: family, friends, neighbours, and other social support systems of older adults living with frailty.

**Health care**: services provided across different settings (e.g., institutional and community), and by different providers (e.g., doctors, nurses, social workers, and occupational and physical therapists) that addresses the full spectrum and diversity of frail older adults’ needs. Note that, for brevity in the review, we have defined health care to include social care.

**Health policy**: setting a direction and providing resources for action to protect, promote, and restore mental and physical well-being, as defined by the Canadian Minister of Justice.

## Appendix 2

**Table 1:** Meeting Statements

Which of the following do you most identify as?^a^
Easy to read information in waiting rooms is an effective way to communicate to patients
If I were a patient, I would read a one-page summary about research projects
TVN needs to play a central role in providing guidance and support for patient engagement in research
We need to educate health care organizations about research and the importance of engagement
We need a flexible engagement model (technology supported/money and time to support participation/go to the grail citizen)
Research is needed into why/how the frail elder becomes engaged in research
Frail elders do not always identify as frail
The voice of the frail elder is not always represented by the voice of the caregiver
We need to consider different venues outside the usual and need for co-presenters (researcher + frail citizen)
We need to consider alternative sources of information
TVN needs to provide resources/experts to researchers that help them to effectively conduct person-centred research
Need research measures/outcomes are that are meaningful to frail citizens (e.g., pain/function/good death/Quality of Life)
Research teams should have a citizen leader in addition to a scientific leader
TVN should support citizens/family caregivers to mentor each other in the research process
TVN needs to train researchers about how to engage frail citizens effectively
Engagement of frail elderly in health and social organizations should be mandated
Continuity of care should be supported by electronic health records and other tools to communicate decisions between care settings
TVN needs to create tools and education packages for health care providers on ways to meaningfully engage frail elderly and caregivers
TVN and advocates of frail elderly issues should make the issue of frailty a priority at local/regional levels so that it can have more political influence

Responses to questions were structured as follows, unless stated otherwise: Rate the extent to which you agree with the below statement. Strongly agree; agree; neutral; disagree; strongly disagree

^a^Which of the following do you most identify as? Advocacy group representative; citizen or volunteer caregiver; health care provider or administrator; policy person; researcher; other
